# Antibiotic dosage prescribed in oral implant surgery: A meta-analysis of cross-sectional surveys

**DOI:** 10.1371/journal.pone.0236981

**Published:** 2020-08-18

**Authors:** Fabio Rodríguez Sánchez, Iciar Arteagoitia, Wim Teughels, Carlos Rodríguez Andrés, Marc Quirynen

**Affiliations:** 1 Department of Preventive Medicine and Public Health, University of the Basque Country, Bilbao, Spain; 2 Department of Oral Health Sciences, Section Periodontology, Catholic University of Leuven & University Hospitals Leuven, Leuven, Belgium; 3 Department of Stomatology, University of the Basque Country, Bilbao, Spain; 4 Bioruces Health Research Institute, Cruces University Hospital, Barakaldo, Spain; Danube Private University, AUSTRIA

## Abstract

This study aimed to assess the dosage and types of antibiotics prescribed in oral implant surgery, compare them among the different subpopulations (country and prescription regimens) and against the evidence-based recommended dosage: a 2-gram single preoperative dose of amoxicillin. A meta-analysis of cross-sectional surveys was conducted, which reports the overall dosage (and type) of antibiotics prescribed in combination with implant placement. PubMed, Cochrane, Science, Direct, and EMBASE via OVID were searched until April 2019. Three reviewers independently undertook data extraction and risk of bias assessment. The outcome variable was set on the average of prophylactic antibiotics prescribed per oral implant surgery. Overall, 726 participants from five cross-sectional surveys, representing five different countries were finally included. Amoxicillin was the most prescribed antibiotic. On average, 10,724 mg of antibiotics were prescribed per implant surgery. This average was significantly (*p<0*.*001*) higher than 2,000 mg. Overall, amoxicillin doses were significantly higher than 2,000 mg (9,700 mg, *p<0*.*001*). All prescribed amoxicillin regimens independently contained more than 2,000 mg, including those comprising only preoperative amoxicillin (2,175 mg, *p = 0*.*006*). Exclusive preoperative antibiotic regimens were the only subgroup with prescription dosages below this threshold (*p = 0*.*091*). Significant variations in antibiotic prescriptions were found among different countries and antibiotic regimens (*p<0*.*001*). In conclusion, the average dose of antibiotics prescribed per oral implant surgery was larger than the evidence-based recommended dose in healthy patients and straightforward conditions. In addition, variations in the average antibiotic dosages were found among different countries and prescription regimens.

## Introduction

Oral implant therapy has developed into a very predictable treatment for the rehabilitation of a partial or complete edentulous oral cavity [1–3]. Nevertheless, oral implant failures do occur [4]. Postoperative infection after bacterial contamination of the surgical site is believed to be one of the main sources of early implant failures; however, it is also known to be associated to certain delayed implant failures [5]. Therefore, perioperative antibiotics have been studied and recommended to prevent these complications [6–11].

Reviews published in this field found that antibiotics were not effective in reducing the incidence of post-operative infections; nonetheless, preoperative antibiotics were found to be beneficial in preventing oral implant failures [8,9,12]. Esposito et al. [9] suggested that routinely prescribing a single pre-operative dose of 2,000 mg of amoxicillin might prevent implant failures in healthy patients and in straightforward conditions. However, 25 patients would need to receive this treatment in order to prevent just one patient from having an implant failure [9].

The prescription of prophylactic antibiotics in oral implant surgery remains controversial [13]. Numerous cross-sectional surveys have been conducted to assess prescription habits in oral implant surgery among dental professionals in different countries [14–26]. These studies reported a wide range of different antibiotic prescriptions and a wide selection of antibiotic types. Recommendations published in recent meta-analyses are often not followed. This emphasizes the need to establish standardized guidelines to support clinicians' decision-making practices [15,22–25].

Irrational use of antibiotics may lead to an unjustified increase in economic costs and adverse reactions such as allergies, toxicity, gastrointestinal disorders and bacterial resistance [27,28]. The latter condition has become a major threat worldwide. Recent studies have shown a direct relationship between antibiotic consumption and the emergence and dissemination of resistant bacterial strains [29].

This alarming situation, coupled with the substantial growth of the oral implant market in recent years [30], predicates an important public health concern. The prescription of antibiotics in dentistry is still rising despite many campaigns to prevent their excessive use [31,32]. Moreover, additional studies have been requested to better assess antibiotic prescription behaviors in dentistry [33]. Consequently, it was deemed necessary to evaluate the prophylactic antibiotic treatments prescribed in oral implant therapy and to determine the quantity of antibiotics that may be considered as overtreatment. As a result, this would permit us to estimate the potential risk caused by the irrational use of prophylactic antibiotics in this situation.

This meta-analysis of cross-sectional surveys primarily aimed to assess the dosage and types of antibiotics prescribed per oral implant surgery. The secondary aim was to contrast the average dosage of prescribed antibiotics against the evidence-based recommended regimen in healthy patients and in straightforward conditions: a single 2-g preoperative dose of amoxicillin [9].

An additional aim of this study was to assess the differences in dosage and antibiotic type between countries and prescription regimens.

The null hypotheses were postulated as follows: (1) the average dosage of prophylactic antibiotics prescribed per oral implant surgery is equal to a single dose of 2,000 mg and (2) there are no variations in the average dosage of prescribed antibiotics among the different countries and prescription regimens.

## Methods

The study was conducted and reported in accordance with the Meta-analysis of Observational Studies in Epidemiology group [34]. Details of the protocol for this meta-analysis were registered on the International Prospective Register of Systematic Reviews (PROSPERO) with the following register identification: CRD42020156885.

Eligible studies included all articles evaluating antibiotic prescriptions in association with oral implant surgery and in adherence with the following Participants; Intervention; Comparison; Outcome and Study type (PICOS) framework:

Participants: General dental practitioners or specialists placing oral implants.

Intervention: Antibiotic prescriptions in association with oral implant surgery.

Comparisons:

1. Evidence-based recommended dosage in healthy patients and in routine conditions: single pre-operative dose of 2,000 mg [9].
2. Comparisons among different subpopulations (countries, antibiotic types and prescription regimens).

Outcomes: Average dosage and types of antibiotics prescribed per oral implant surgery.

Study type: Cross-sectional survey.

Publications were excluded if they were clinical trials, case series or retrospective studies. There were no restrictions on language or publication year. Publications that did not report enough information to calculate the total dosage of antibiotics contained in their participants’ prescriptions were also excluded.

Searches were conducted in the following electronic databases up to June 4, 2020: Embase, PubMed, Ovid Medline, Scopus, Science-Direct, Web of Knowledge, as well as the Spanish General University Board database of doctoral theses in Spain, the Spanish National Research Council bibliographic databases, and the Spanish Medical Index.

Three independent investigators carried out the search in the databases. The searched terms were descriptors of the PICO components: antibiotics, oral implant surgery, dental implant surgery, oral implant placement, dental implant placement, and cross-sectional survey.

MeSH and search algorithms connected with Boolean operators were used as keywords for the electronic search. No filters were applied in the Ovid Medline and PubMed search: (antibiotic) AND (((oral OR dental) implant AND surgery) OR ((oral OR dental) implant AND placement)) AND (survey). In Scopus, the search was limited to “Dentistry” and “Article” for subject area and document type: (antibiotic) AND (((oral OR dental) implant AND surgery) OR ((oral OR dental) implant AND placement)) AND (survey) AND (LIMIT-TO (DOCTYPE, "ar")) AND (LIMIT-TO (SUBJAREA, "DENT")). The search in In Web of Knowledge was filtered by “Article”: TS = (antibiotic "AND" oral implant surgery "OR" dental implant surgery "AND" survey). In Science Direct, “Research articles” filtered the search: (antibiotic) AND (((oral OR dental) implant AND surgery) OR ((oral OR dental) implant AND placement)) AND (survey).

The search in Embase was limited to “Article”, “Short Survey”, “Article in Press” and “Questionnaire”: (antibiotic) AND (((oral OR dental) implant AND surgery) OR ((oral OR dental) implant AND placement)) AND (survey) AND ('article'/it OR 'article in press'/it OR 'short survey'/it) AND 'questionnaire'/de.

For databases in Spanish, the following terms were used: (antibioticos) AND (implante dental O implante oral) AND (encuesta).

The references of all retrieved papers were reviewed as well. No potentially unpublished material could be identified.

**Fig 1:**
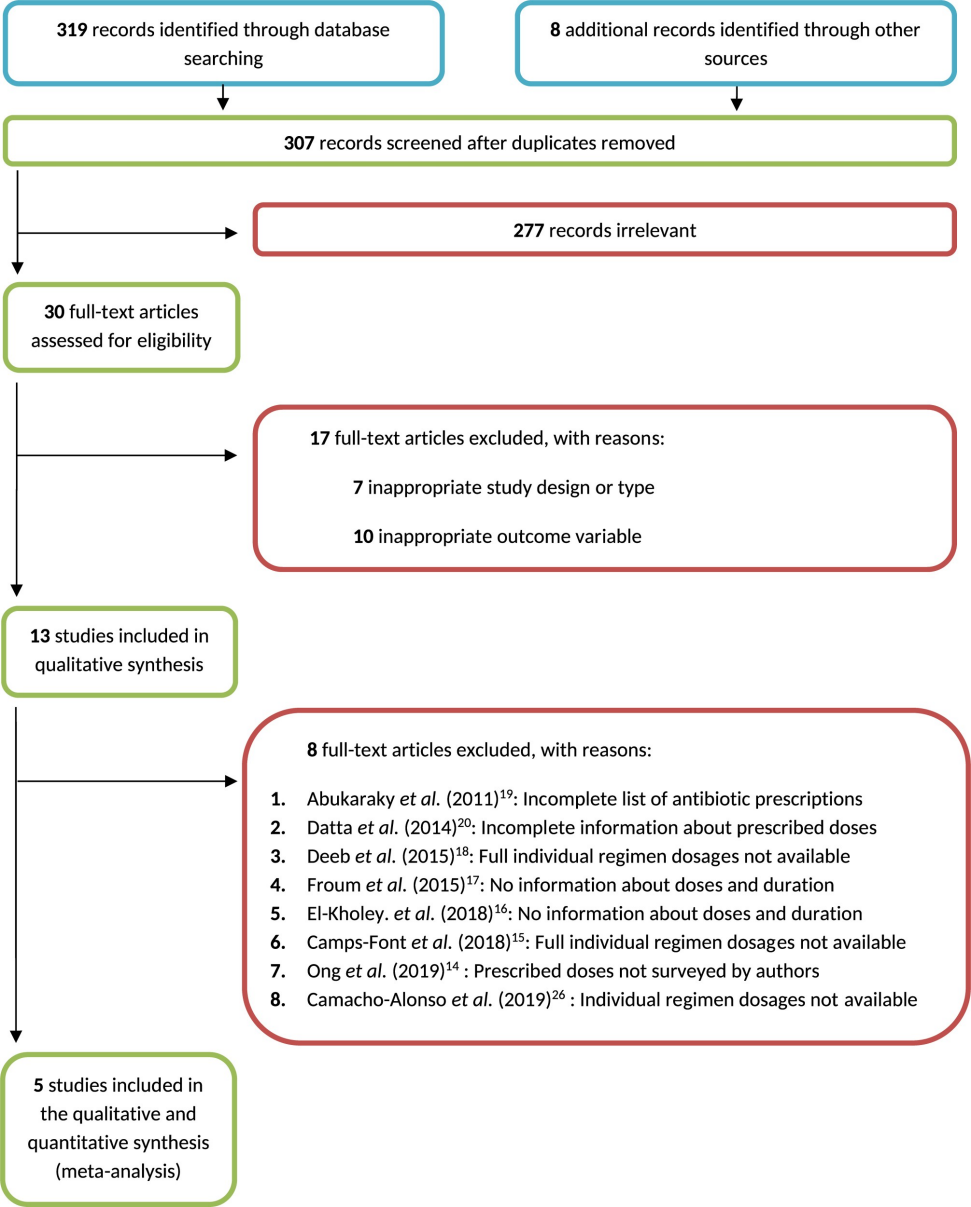
Flow diagram. This diagram describes the study selection process.

Two independent reviewers (F.R.S. and C.R.A.) screened the titles and abstracts from the records identified from the search by using Cochrane's online software [35]. Full-text articles were acquired for records that fulfilled the inclusion criteria. The researchers contacted every corresponding author when extra information was required in the selection process. All discrepancies were discussed with a third researcher (I.A.). The reasons for exclusion were reported (Fig 1).

The recorded data included the following: antibiotic type, regimen (preoperative, postoperative or both), dose, treatment duration and country. If the original dataset of an included study could not be obtained, information relating to the antibiotic type, prophylactic regimen (preoperative, postoperative or both), dose and treatment duration were extracted from the published paper by two independent researchers (F.R.S. and C.R.A.). A third party was consulted to resolve any disagreement (I.A.). Calculations using data from tables were performed if the data on any variable were not explicitly stated in the text. The corresponding authors of 8 different studies were contacted because the necessary information from their studies were unclear [14–20,26].

One study surveyed 133 Swedish dental professionals [21]. Of these, 98 prescribed antibiotics while 35 did not prescribe any prophylactic antibiotics. This study completely described 85 antibiotic regimens; however, there were unfortunately 13 missing antibiotic regimens. After contacting the authors, no extra information was obtained. Therefore, the 85 dentists who prescribed antibiotics were included with a proportionate number of non-prescribing professionals (n = 22) in place of the 35 at the beginning.

The same adjustment was applied to other included studies with 29 participants who were unfortunately excluded because they did not provide a description of their prescription regimens (14 from Spain, 6 from Italy and 9 from the Netherlands). The newly calculated and proportionate numbers of non-prescribing professionals in these cases were 3.75, 0.96 and 4.7 respectively, while the original numbers were 4, 1 and 5 respectively. As the calculated values were very close to the original ones, it was decided to keep the initial numbers in order to perform the most conservative analysis possible [24–26].

The authors of the other five articles were unsuccessfully contacted in order to collect necessary data for inclusion in the meta-analysis [15,17–19]. The authors of two articles were successfully contacted; however, data requested on prescription dosage was insufficient for inclusion in the meta-analysis because their surveys did not collect this information [14,20].

Two independent reviewers (F.R.S. and C.R.A.) assessed the quality of the included studies using the National Heart, Lung, and Blood Institute Quality Assessment Tool for Observational Cohort and Cross-Sectional Studies [36]. All discrepancies were discussed with a third researcher (I.A.). The studies were categorized as low, moderate or high quality if the percentage of affirmative answers to the checklist was less than 50%, between 50% and 80% or more than 80% respectively.

Each included study presented different datasets and data codifications. This heterogeneous presentation of data was for a limitation to performing a proper quantitative analysis (meta-analysis). To overcome this limitation and accomplish the study objectives, a uniform database with the original dataset from each study was created. The software STATA version 15 (StataCorp LLC, College Station, TX, USA) was used to generate this database and to perform all statistical analyses.

The average dosage (mg) of prophylactic antibiotics prescribed per implant surgery was calculated according to the individual prescription regimens (multiplying the treatment dose, dosage and the corresponding duration) with an estimation of the standard deviation (SD). Participants who never prescribe prophylactic antibiotics for oral implant surgery were also included in this analysis. The normal distribution of the outcome data was graphically assessed using quantile-quantile plots (Q-Q plots).

Student’s t-test was used to compare the means of the prophylactic antibiotics prescribed per study, country and prescription regimen against the evidence-based recommended regimen: single pre-operative dose of 2,000 mg. In this analysis, prescriptions were included only if they contained antibiotics with a Defined Daily Dose (DDD) equal to the evidence-based recommended regimen (2,000 mg) or equal to the DDD of amoxicillin (1,500 mg) according to the Anatomical Therapeutic Chemical system of the World Health Organization [37].

Multiple f-tests were used to compare the variations in different groups. Depending on the variance analysis, multiple t-tests for equal or unequal variances were performed to compare the means of the antibiotics prescribed in the included studies. Bonferroni standard corrections were performed in both, f- and t-tests. In both tests, the α-value was calculated by dividing 0.05 by the total number of performed comparisons.

## Results

Five cross-sectional surveys were finally included in this meta-analysis [21–25]. Table 1 shows the descriptive information for of each study included in the quantitative analysis. A flow chart describes the selection process, records and full-text exclusions with their justifications (Fig 1).

**Table 1:** Descriptive information of each included study.

Study (year)	Country	n	Type of professionals	Most frequently prescribed regimen (n)	Participants routinely prescribing prophylactic antibiotics (n)
*Khalil et al., (2012)[21]*	Sweden	133	General dentists	2 g of oral amoxicillin pre-operatively (27)	74% (98)
*Ireland et al., (2012)[22]*	United Kingdom	109	General dentists	3 g of oral amoxicillin one hour pre-operatively (54)	72% (76)
*Arteagoitia et al., (2018)[23]*	Spain	233	General dentists	500 mg of oral amoxicillin TID 1 day pre-operatively and for 7 days post-operatively (10)	89% (207)
*Rodríguez Sánchez et al., (2019)[24]*	Netherlands	151	General dentists, oral implantologists, periodontists and maxillofacial surgeons	2 g of oral amoxicillin 1 hour or immediately prior to surgery (35)	44% (66)
*Rodríguez Sánchez et al., (2019)[25]*	Italy	160	General dentists and oral surgeons	875/125 mg of oral amoxicillin/clavulanic acid BID 1 day pre-operatively and for 5 days post-operatively (15)	84% (134)

BID: Two times daily; TID: Three times daily.

Four studies were judged as being of moderate quality [21–25] and one of low quality [22]. The percentage of affirmative answers to the National Health Index checklist was 75% for the study performed in Sweden, 54.5% for the other 3 studies (Spain, the Netherlands and Italy) and 45.5% for the study performed in the United Kingdom. The data distribution of the outcome variable is shown in the Q–Q plots (S1 Fig).

**Fig 2:**
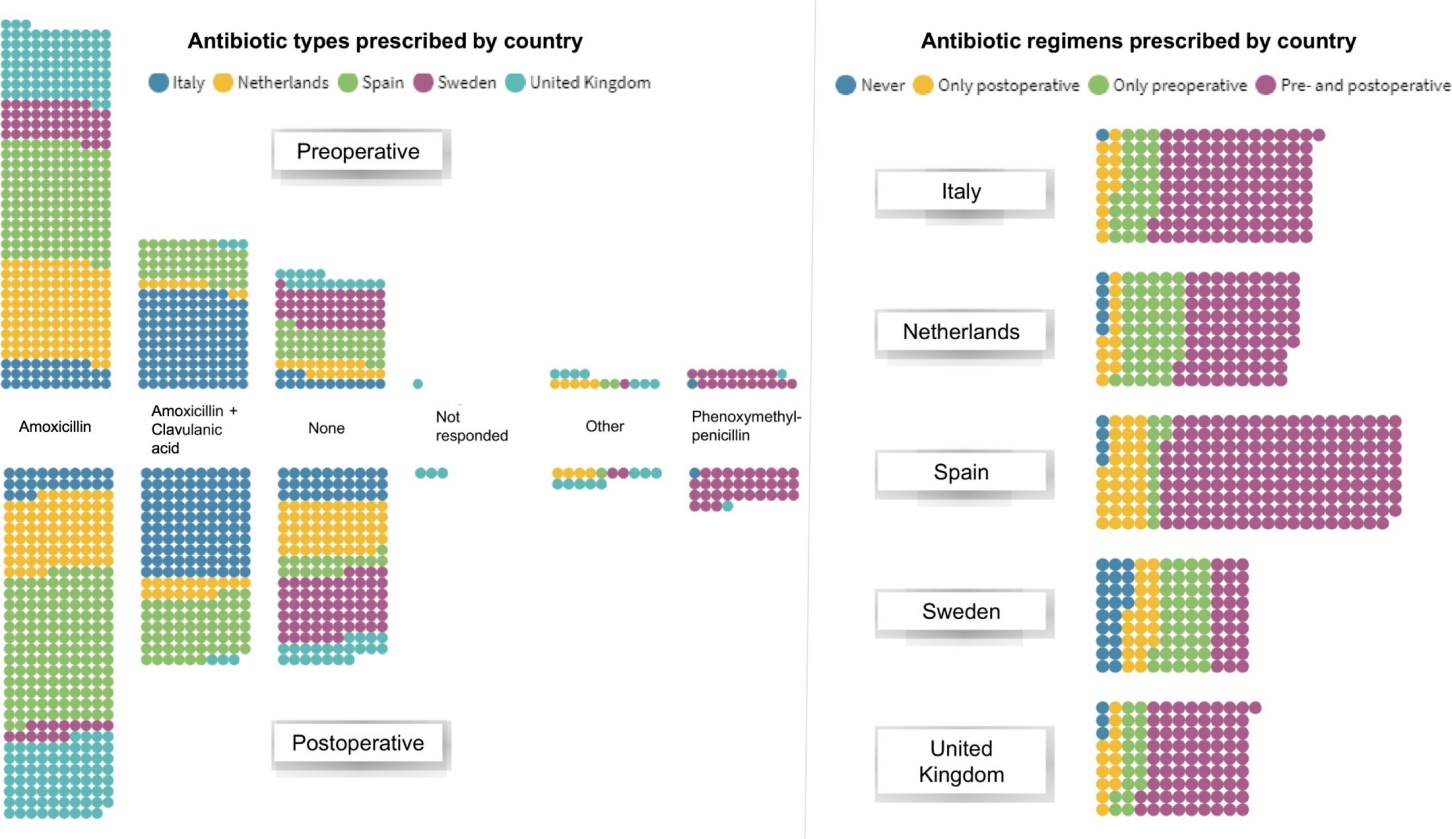
Antibiotic types and regimens prescribed per country. Each dot represents one participant included in the meta-analysis.

Overall, 726 participants were enrolled in this meta-analysis. All prophylactic prescriptions consisted of oral antibiotics. Fig 2 illustrates the antibiotic types and regimens prescribed per country (Fig 2).

On average, 10,724 mg of prophylactic antibiotics were prescribed per oral implant surgery. This average dose of antibiotics was found to be significantly higher (*p<0*.*001*) than the evidence-based recommended dose (2,000 mg).

Table 2 shows the average dose of prophylactic antibiotics prescribed per antibiotic type and country. Amoxicillin was the most frequently prescribed antibiotic type, followed by amoxicillin in association with clavulanic acid. Most professionals from the Italian survey, followed by the participants from the Spanish survey prescribed clavulanic acid (Table 2).

**Table 2:** Average dosage of prophylactic antibiotics (mg) prescribed per country and antibiotic type.

*Antibiotic type / Country*		Spain	Italy	Netherlands	Sweden	United Kingdom	Overall	ATC code	DDD
**Amoxicillin**	Mean	1,5047	8,672	6,561	4,642	7,399	9,700	J01CA04	1,500
	*SD*	*6*,*829*	*5*,*180*	*4*,*207*	*5*,*325*	*3*,*676*	*6*,*726*		
	n	150	32	111	44	86	423		
**Amoxicillin / Clavulanic Acid**	Mean	19,178	10,685	7,600	-	17,494	13,208	J01CR02	1,500
	*SD*	*8*,*228*	*4*,*839*	*4*,*029*	-	*14*,*946*	*7*,*472*		
	n	56	117	10	0	4	187		
**Penicillin V**	Mean	-	15,000	-	18,079	3,000	17,625	J01CE02	2,000
	*SD*	-	0	-	*17*,*197*	0	*16*,*925*		
	n	0	1	0	38	1	40		
**Amoxicillin / Amoxicillin + Clavulanic Acid**	Mean	25,166	11,000	10,296	-	8,812	13,031	J01CA04 / J01CR02	1,500 / 1,500
	*SD*	*763*	*7550*	*1*,*406*	-	*265*	6,726		
	n	3	3	8	0	2	16		
**Azithromycin**	Mean	-	-	11,000	-	10,100	10,550	J01FA10	300
	*SD*	-	-	*3*,*869*	-	*1*,*732*	*2*,*726*		
	n	0	0	3	0	3	6		
**Clindamycin**	Mean	-	-	11,000	600	12,600	6,600	J01FF01	1,200
	*SD*	-	-	*3*,*869*	*0*	*0*	*6*,*600*		
	n	0	0	1	1	1	3		
**Clindamycin / Amoxicillin + Clavulanic Acid**	Mean	-	-	11,200	-	-	11,200	J01FF01 / J01CR02	1,200 / 1,500
	*SD*	-	-	*2*,*687*	-	-	*2*,*687*		
	n	0	0	2	0	0	2		
**Amoxicillin / Penicillin V**	Mean	-	-	-	24,000	8,000	16,000	J01CA04 / J01CE02	1,500 / 2,000
	*SD*	-	-	-	*0*	*0*	*11*,*314*		
	n	0	0	0	1	1	2		
**Metronidazole**	Mean	-	-	-	6,000	25,200	15,600	J01XD01	1,500
	*SD*	-	-	-	-	*0*	*13*,*576*		
	n	0	0	0	1	1	2		
**Erythromycin**	Mean	3,000	-	-	-	6,500	4,750	J01FA01	2,000
	*SD*	0	-	-	-	*0*	*2*,*475*		
	n	1	0	0	0	1	2		
**Amoxicillin / Metronidazole**	Mean	-	-	-	-	24,000	24,000	J01CA04 / J01XD01	1,500 / 1,500
	*SD*	-	-	-	-	*0*	*0*		
	n	0	0	0	0	1	1		
**Primcillin**	Mean	-	-	-	-	18,400	18,400	J01CE02	2,000
	*SD*	-	-	-	-	*0*	*0*		
	n	0	0	0	0	1	1		
**Cefazolin**	Mean	-	-	-	-	8,250	8,250	J01DC02	3,000
	*SD*	-	-	-	-	*0*	*0*		
	n	0	0	0	0	1	1		
**Cefuroxime / Amoxicillin + Clavulanic Acid**	Mean	-	-	-	-	14,375	14,375	J01DC04 / J01CR02	500 / 1,500
	*SD*	-	-	-	-	*0*	*0*		
	n	0	0	0	0	1	1		
**Cefazolin / Amoxicillin + Clavulanic Acid**	Mean	25,000	-	-	-	-	25,000	J01DB04 / J01CR02	3,000 / 1,500
	*SD*	0	-	-	-	-	*0*		
	n	1	0	0	0	0	1		
**Not responded**	Mean	-	-	2,000	-	10,500	7,667	-	-
	*SD*	-	-	0	-	0	4,907		
	n	0	0	1	0	2	3		
**None**	Mean	0	0	0	0	0	0	-	-
	*SD*	*0*	*0*	*0*	*0*	*0*	*0*		
	n	4	1	5	22	3	35		
**Overall**	Mean	15,974	10,231	6,742	8,615	8,216	10,713	-	-
	*SD*	*7*,*764*	*5*,*044*	*4*,*310*	*13*,*103*	*5*,*426*	*8*,*315*		
	n	215	154	141	107	109	726		

The name Penicillin V was used in this table instead of Phenoxymethylpenicillin, being both different names for the same drug.

SD: standard deviation; DDD: defined daily dose; ATC: Anatomical Therapeutic Chemical

The overall dose of the prescribed amoxicillin was significantly higher than 2,000 mg (9,700 mg, *p<0*.*001*). All the regimens with only amoxicillin independently comprised a significantly higher dose than the reference of 2,000 mg, including those with only pre-operative amoxicillin (2,175 mg, *p = 0*.*006*). Nevertheless, the participants from United Kingdom prescribing exclusively pre-operative amoxicillin were the only ones that significantly (*p<0*.*001)* did it above the level of 2,000 mg per oral implant surgery (Table 3).

**Table 3:** Average dosage of amoxicillin (mg) prescribed per country and prescription regimen.

*Prescription regimen / Country*		Spain	Italy	Netherlands	Sweden	United Kingdom	Overall
**Only pre-operative**	Mean	2,182†	1,900‡	2,042¶	2,025††	2,926*	2,175‡‡
	*SD*	*1*,*401*	*316*	*462*	*211*	*528*	*655*
	n	11	10	42	30	17	110
**Only post-operative**	Mean	13,433	1,0667	9,300	-	6,675	10,769*
	*SD*	*4*,*603*	*2*,*309*	*1*,*549*	-	*1*,*390*	*4*,*345*
	n	21	3	10	0	10	44
**Pre- & post-operative**	Mean	16,534	11,921	9,314	10,250	8,810	12,603*
	*SD*	*6*,*111*	*2*,*878*	*3*,*042*	*6*,*635*	*3*,*384*	*6*,*012*
	n	118	19	59	14	59	269
**Overall**	Mean	15,047*	8,672*	6,561*	4,642**	7,399*	9,700*
	*SD*	*6*,*829*	*5*,*180*	*4*,*207*	*5*,*325*	*3*,*676*	*6*,*726*
	n	150	32	111	44	86	423

Bilateral T-test contrasting mean = 2,000 mg

*p<0.*001*

**p = 0.*002*

†*p = 0*.*676*

‡*p = 0*.*343*

¶*p = 0*.*561*

††*p = 0*.*521;*

‡‡*p = 0*.*006*

SD: Standard deviation

**Fig 3:**
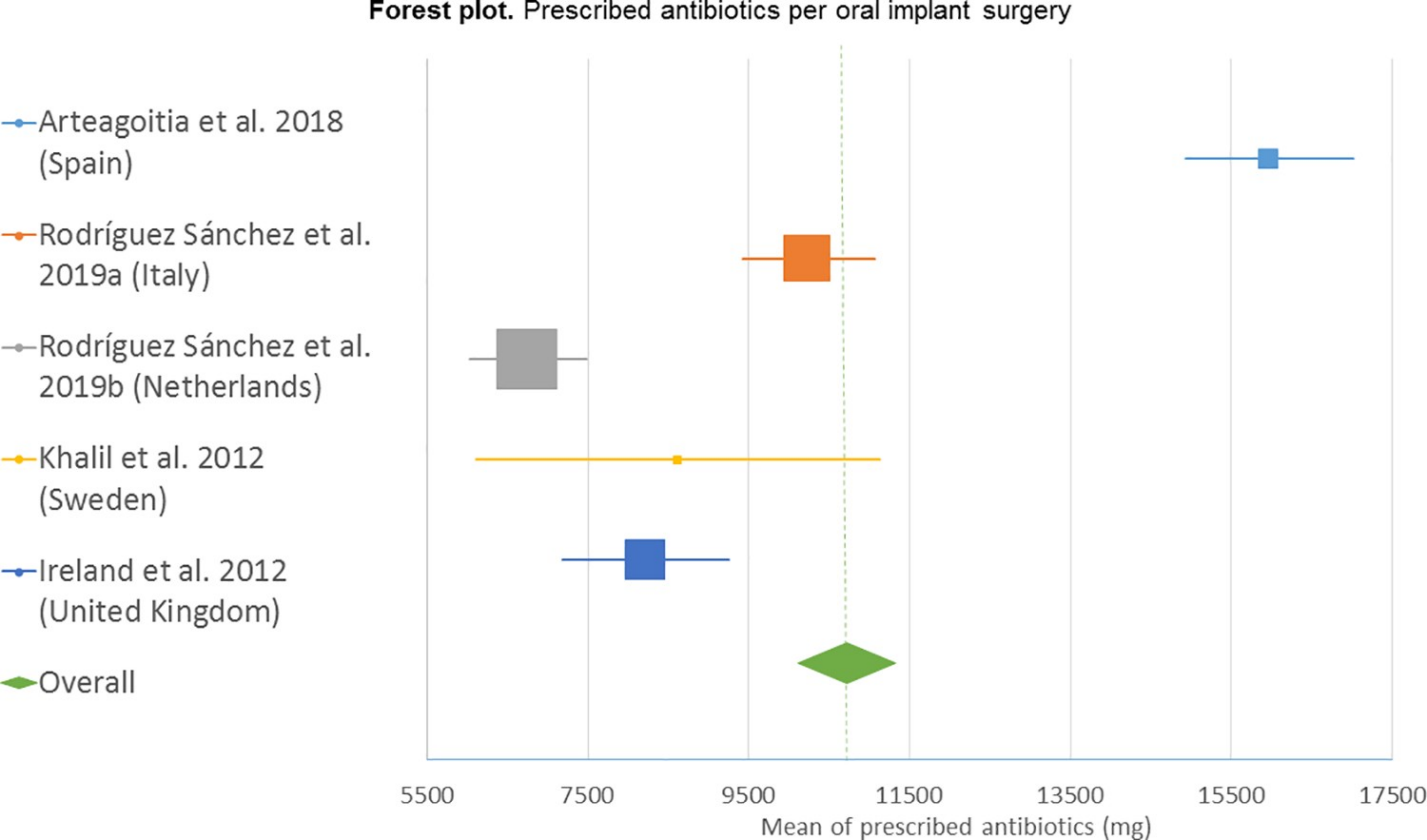
Forest plot. The forest plot represents the estimates of the mean values and 95% confidence intervals for each outcome variable. The area of the squares around the mean values is proportional to the weight of the study in the analysis. A continuous horizontal line indicates the 95% confidence intervals, while a rhombus and a dotted line indicate the overall mean value.

Among the different subpopulations (country and prescription regimen), professionals prescribing only pre-operative antibiotics were the only ones whose antibiotic prescriptions (2,110 mg) were not significantly (*p = 0*.*091*) above this threshold (Table 4). A forest plot taking the outcome variable into account is shown in Fig 3 (Fig 3).

**Table 4:** Average dose of prophylactic antibiotics (mg) prescribed per country and prescription regimen.

*Prescription regimen / Country*		Spain	Italy	Netherlands	Sweden	United Kingdom	Overall
**Never**	Mean	-	-	-	-	-	-
	*SD*	-	-	-	-	-	-
	n	4	1	5	22	3	35
**Only pre-operative**	Mean	2,182**	1,786††	2,037‡‡	2,020¶	2,930*	2,110¶¶
	*SD*	*1*,*401*	*630*	*451*	*302*	*513*	*676*
	n	11	28	44	37	18	138
**Only post-operative**	Mean	13,210	10,404	9,156	31,600	6,579	15,593*
	*SD*	*5*,*988*	*2*,*440*	*1*,*495*	*13*,*003*	*1*,*356*	*11*,*490*
	n	32	13	12	20	11	88
**Pre- & post-operative**	Mean	17,830	12,414	9,413	7,327	9,992	13,282*
	*SD*	*6*,*782*	*3*,*254*	*2*,*937*	*5*,*770*	*5*,*672*	*6*,*480*
	n	166	112	73	26	67	444
**Overall**	Mean	15,993*	10,231*	6,617*	8,545*	8,025*	10,724*
	*SD*	*7*,*725*	*5*,*044*	*4*,*287*	*13*,*119*	*5*,*614*	*8*,*377*
	n	213	154	134	105†	99	705‡

† 13 participants with missing regimens could not be included. To keep a proportional number of non-prescribing participants, only 22 out of the original 35 participants who never prescribe prophylactic antibiotics were included.

‡ 21 participants excluded because their prescriptions included antibiotic types with DDDs different to 2,000 mg or from the DDD value of amoxicillin (1,500 mg).

Bilateral T-test contrasting mean = 2,000 mg

*p<0.*001*

**p<0.*676*

††*p = 0*.*083*

‡‡*p = 0*.*590*

¶*p = 0*.*781*

¶¶*p = 0*.*091*

SD: standard deviation

Bartlett's test was found to be statistically significant (*p<0*.*001*) among the different countries and prophylactic prescription regimens. Moreover, I^2^ was found to be low (18.7%). Therefore, low heterogeneity was found between countries (Table 5).

**Table 5:** Multiple comparison of means and variances of prescribed prophylactic antibiotics (mg).

Group comparisons	Contrast of means†	95% CI	*P*-value‡	*p*-value§
*Spain vs*. *Italy*	5,743	4,430–7,056	<0.001	<0.001
*Spain vs*. *Netherlands*	9,232	7,969–10,495	<0.001	<0.001
*Italy vs*. *Netherlands*	3,489	2,409–4,569	0.058	<0.001
*Spain vs*. *Sweden*	7,436	4,740–1,032	<0.001	<0.001
*Italy vs*. *Sweden*	1,693	-922–4,307	<0.001	0.202
*Netherlands vs*. *Sweden*	-1,796	-4,386–794	<0.001	0.172
*Spain vs*. *United Kingdom*	7,758	6,298–9,219	<0.001	<0.001
*Italy vs*. *United Kingdom*	2,015	732–3,298	0.405	0.002
*United Kingdom vs*. *Netherlands*	1,473	261–2,686	0.011	0.017
*Sweden vs*. *United Kingdom*	323	-2,367–3,012	<0.001	0.813
*Pre- & post-operative vs*. *Only pre-operative*	11,022	10,402–11,641	<0.001	<0.001
*Only post-operative vs*. *Pre- & post-operative*	2,122	-329–4,573	<0.001	0.089
*Only pre-operative vs*. *Only post-operative*	13,144	10,756–15,531	<0.001	<0.001

† Differences were calculated by deducting the mean value in the second group from that of the first.

‡ Bilateral F-tests contrasting H_0_: equal variances. The α-value was calculated by dividing 0.05 by the total number of performed comparisons, 10 when comparing countries (α-value = 0.005) and 3 when comparing prescription regimens (α-value = 0.016)

§ Two-sample t-test contrasting means with equal or unequal variances depending on the variances F-tests. The α-value was calculated by dividing 0.05 by the total number of performed comparisons: 10 when comparing countries (α-value = 0.005) and 3 when comparing prescription regimens (α-value = 0.016)

CI: confidence interval.

The multiple-comparison analysis of variances showed that all comparisons of variances were statistically significant, except for three: Italy against the Netherlands, Italy against the United Kingdom, and the United Kingdom against the Netherlands. Therefore, both countries in each of these comparisons were found to be homogeneous, relating to the dosages of prescribed antibiotics.

In addition, mean comparisons were found to be statistically significant, except for Italy against Sweden, the Netherlands against Sweden, the United Kingdom against the Netherlands, Sweden against the United Kingdom and only post-operative against pre- and postoperative. Consequently, both countries in each of these comparisons were found to prescribe a similar average dosage of prophylactic antibiotics (Table 5).

## Discussion

This meta-analysis quantitatively assessed the prescriptions of prophylactic antibiotics in association with oral implant surgery and compared them to the existing scientific recommendations. In addition, this study provides quantitative comparisons of the average dosage of antibiotics and the regimens prescribed in oral implant surgery by professionals from different countries.

This meta-analysis indicates that the average dosage of prophylactic antibiotics prescribed in conjunction with oral implant surgery is approximately five times larger than the evidence-based recommendations for healthy patients and straightforward conditions: a 2-gram single preoperative dose. Even for prescriptions of only pre-operative antibiotics, the average dosage was higher than the evidence-based recommended dose [9]. Countries presented great variability in their average dosage of prescribed antibiotics and prescription regimens. These findings may be the consequence of a lack of consensus on the use of antibiotics in oral implant surgery among clinicians. Furthermore, the prescription variances found among the different countries included in this meta-analysis may be attributed to this clinician’s disagreement coupled with the idiosyncratic and cultural prescription habits of each country.

Cross-sectional studies may be the most appropriate study design to estimate the antibiotics prescribed in oral implant surgery, due to the lack of official records. Nevertheless, participants’ statements in this kind of study may differ from their real behavior and the included participants may have changed their conduct over time, since the included surveys were performed over the past years. In addition, patient interviews are required to measure the real drug intake at the patient level because they do not always follow the prescriptions.

Despite all the efforts made to include the largest number of cross-sectional surveys in this meta-analysis, only five studies from five countries could be included. Moreover, the cross-sectional surveys did not reach all practitioners placing oral implants in each country, which may represent a source of bias. The combined analysis of all included studies in this meta-analysis increased the sample size and consequently, the power of the planned hypothesis analysis. The variability found among the surveys did not cause heterogeneity in the results. The quality analysis performed through each of the included surveys suggests that the quality of this meta-analysis may be moderate, which could represent an important limitation. Consequently, the findings of this study must be considered cautiously due to the inherent limitations of any cross-sectional survey and the intrinsic weakness of the included papers, coupled with the limitations of this meta-analysis. These facts must be contemplated with utmost care to correctly interpret the outcomes of this meta-analysis.

Regardless of the determination of the authors, not all participants of the included surveys could be enrolled in this meta-analysis because of missing information. This may represent only a minor limitation in the data collection procedure as this problem was later solved by including a proportionated sample of non-prescribing professionals.

The average dosage of prescribed antibiotics was compared against a single pre-operative dose of 2,000 mg, which was considered the evidence-based recommendation in healthy patients and straightforward conditions despite its relative effectiveness [9]. This recommended dose was suggested for amoxicillin; however, but other antibiotic types have different assumed maintenance dosages for their main indications for adults. This could represent significant limitation when contrasting the prescriptions against this recommendation, despite the fact that most majority of the prescriptions included in this meta-analysis involved amoxicillin with or without clavulanic acid or antibiotic types coming from the family of penicillin.

Therefore, only antibiotics types with equal DDDs to amoxicillin or the evidence-based recommendations were included in this comparison. The DDD is the assumed average maintenance dose per day for a drug used for its main indication in adults. The DDDs for anti-infectives are the main rule based on their use in infections of moderate severity [37].

In addition, two cross-sectional surveys (Sweden and the United Kingdom) were performed before these recommendations were published [21,22]. The time lapse since the publication of these studies may have increased the possibilities of changes in the participants' antibiotic prescription habits for oral implant surgery. This means that the current prescriptions could have varied over time and, therefore, the results of this study should be considered cautiously.

The professionals included in this study may present differences in their makeup, with possible variations in the proportion of specialists and general dentists between each country. This could lead to the presence of longer and more frequent antibiotic prescriptions among participants depending on their degree of specialization and the complexity of the surgeries performed.

Nonetheless, three of the cross-sectional surveys, comprising the majority of the participants included in this meta-analysis (70%), contained prescriptions exclusively made for oral implant surgery in healthy patients and straightforward conditions [23–25]. Although the other two surveys may have included some prescriptions based on different circumstances, the majority of their participants (72% and 74% respectively) reported that they routinely prescribed antibiotics regardless of any specific conditions [21,22]. Despite these limitations, the lack of a clinical consensus, rather than the performance of complex surgeries or in patients with compromised health, is most likely the reason for the large differences found between prescribed antibiotics and scientific recommendations.

The findings reported by this meta-analysis suggest that an important number of antibiotic prescriptions might not be based on scientific evidence. This situation may unreasonably increase the risk of adverse events such as allergies, toxicity, gastrointestinal disorders and the development of bacterial resistance [27,28]. This last consequence must be regarded as an extraordinary concern as drug-resistant diseases already cause at least 700,000 deaths a year worldwide [38]. In the most alarming scenario, this figure could rise to 10 million deaths a year by 2050 if no action is taken. The economic damage caused by uncontrolled antimicrobial resistance could be devastating, as it could drive 24 million people into extreme poverty [38]. Moreover, the economic cost of antibiotic prophylaxis for an individual is low but the potential costs for the healthcare system may be substantial and definitely groundless if they are made through irrational prescriptions [39].

Consequently, this study might reveal clinically relevant information for professionals placing oral implants in order to increase their adherence to recommendations when prescribing prophylactic antibiotics and preventing their misuse. The present meta-analysis should inspire new clinical research to improve the guidelines on this topic. This study could also encourage the dissemination of methodologically strong evidence-based guidelines over antibiotic prophylaxis in oral implant surgery, as this may induce behavioral changes in professionals that may eventually correct their prescription patterns [40].

## Conclusions

In conclusion, the average dose of antibiotics prescribed per oral implant surgery was higher than that of the evidence-based recommended regimen in healthy patients and in straightforward conditions. Additionally, there were variances in the average dose of prescribed antibiotics among different countries and prescription regimens.

## Supporting information

S1 Checklist MOOSE (Meta-analyses Of Observational Studies in Epidemiology) checklist. (PDF)

S2 Checklist PRISMA 2009 checklist. (DOC)

S1 Fig Q-Q plots. A dot on the plot corresponds to one of the quantiles of the outcome data distribution (y-coordinate) plotted against the same quantile of the normal distribution (x-coordinate). *Antibiotic types in which DDD is equal to the evidence-based recommended regimen (2,000 mg) or to the DDD of amoxicillin (1,500 mg).
(TIF)

S1 Dataset (XLSX)
