# Supplementary figures and images for: Antibiotic dosage prescribed in oral implant surgery: A meta-analysis of cross-sectional surveys
Source: PLoS One. 2020 Aug 18;15(8):e0236981. doi: 10.1371/journal.pone.0236981 (PMC7446810; doi:10.1371/journal.pone.0236981)

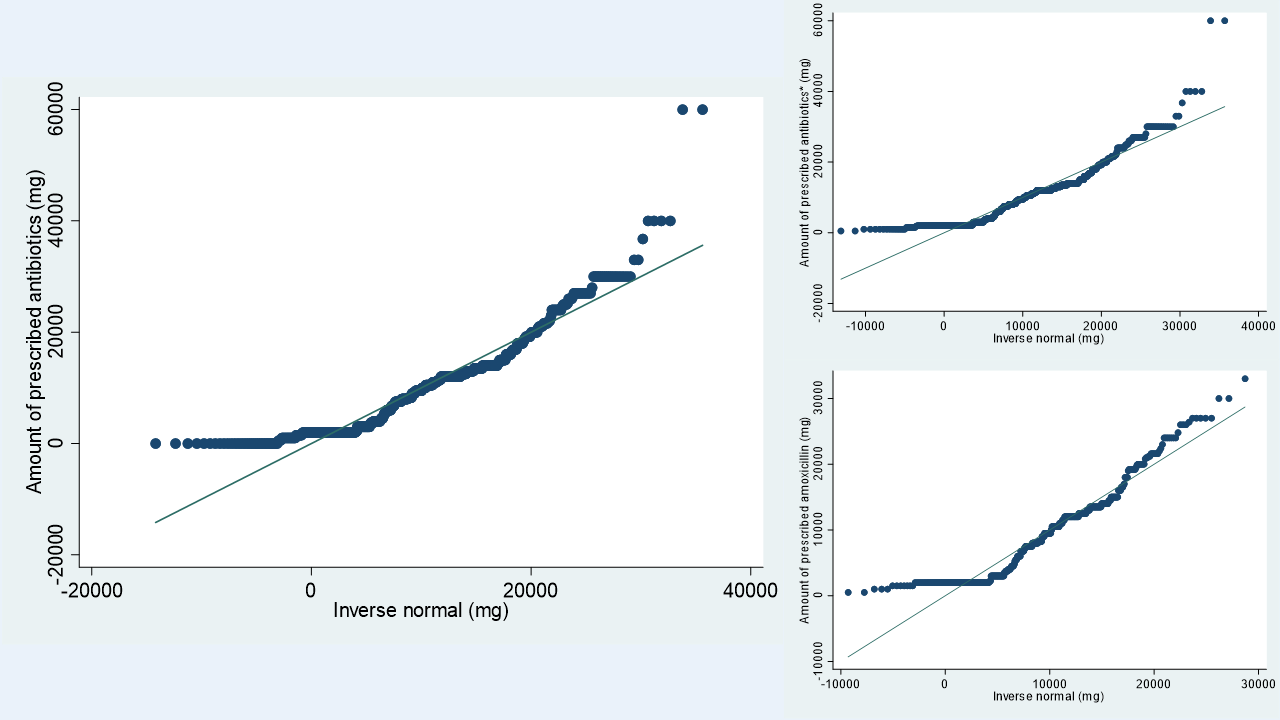

Supplement: S1 Fig — A dot on the plot corresponds to one of the quantiles of the outcome data distribution (y-coordinate) plotted against the same quantile of the normal distribution (x-coordinate). *Antibiotic types in which DDD is equal to the evidence-based recommended regimen (2,000 mg) or to the DDD of amoxicillin (1,500 mg). (TIF) [file pone.0236981.s003.tif]
